# Effect of understaging on local recurrence of rectal cancer

**DOI:** 10.1002/jso.26111

**Published:** 2020-07-11

**Authors:** Louis J. X. Giesen, Wernard A. A. Borstlap, Willem A. Bemelman, Pieter J. Tanis, Cornelis Verhoef, Pim B. Olthof, DUTCH SNAPSHOT RESEARCH GROUP

**Affiliations:** ^1^ Department of Surgical Oncology Erasmus MC Cancer Institute Rotterdam The Netherlands; ^2^ Department of Surgery, Amsterdam UMC, Cancer Centre Amsterdam University of Amsterdam Amsterdam The Netherlands

**Keywords:** colorectal surgery, neoadjuvant therapy, neoplasm staging, rectal neoplasms

## Abstract

**Background and Objectives:**

Magnetic resonance imaging of the pelvis has a limited accuracy to detect positive lymph nodes but does dictate neoadjuvant treatment in rectal cancer. This study aimed to investigate preoperative lymph node understaging and its effects on postoperative local recurrence rate.

**Methods:**

Patients were selected from a retrospective cross‐sectional snapshot study. Patients with emergency surgery, cM1 disease, or unknown cN‐ or (y)pN category were excluded. Clinical and pathologic N‐categories were compared and the impact on local recurrence was determined by multivariable analysis.

**Results:**

Out of 1548 included patients, 233 had preoperatively underestimated lymph node staging based on (y)pN category. Out of the 695 patients staged cN0, 168 (24%) had positive lymph nodes at pathology, and out of the 594 patients staged cN1, 65 (11%) were (y)pN2. Overall 3‐year local recurrence rate was 5%. Clinical N‐category was not associated with local recurrence when corrected for pT‐category, neoadjuvant therapy, and resection margin, neither in patients with (y)pN1 (hazard ratio [HR]: 1.67 (95% confidence interval [CI]: 0.68‐4.12) *P* = .263) nor (y)pN2‐category (HR: 1.91 95% CI: [0.75‐4.84], *P* = .175).

**Conclusion:**

Preoperative understaging of nodal status in rectal cancer is not uncommon. No significant effect on local recurrence or overall survival rates were found in the present study.

## 1 INTRODUCTION

Over the past decades, total mesorectal excision (TME) and neoadjuvant therapy have been associated with reduced local recurrence rates in patients undergoing surgery for rectal cancer.^1^, ^2^, ^3^ With improved quality of TME surgery over time, the absolute contribution of radiotherapy to locoregional control has diminished, especially in early‐stage cancers.^4^ For this reason, indications for neoadjuvant therapy in patients intentionally undergoing TME surgery have changed over time, with improved balance between potential oncological benefit and radiotherapy related morbidity.^5^

Preoperative locoregional staging of rectal cancer is currently performed using magnetic resonance imaging (MRI), and these results dictate the neoadjuvant treatment regimen.^6^, ^7^, ^8^, ^9^ The large randomized trials on neoadjuvant therapy regimens did not routinely use MRI in the preoperative staging, and inclusion was often based on tumor fixity as determined by digital rectal examination.^10^ MRI has made substantial contributions to optimize patient selection and to tailor treatment.^11^ However, preoperative clinical staging using MRI has some restrictions, especially accuracy for suspected positive lymph nodes is limited.^9^, ^12^, ^13^ Furthermore, since organ preserving surgery and nonsurgical management for rectal cancer are gaining prominence, adequate staging might become even more important in the future.

This study aimed to investigate the number of patients who underwent surgical resection for rectal cancer with a preoperative underestimated lymph node category and to analyze the effects of nodal understaging on local recurrence rates.

## 2 METHODS

All data were obtained from the Dutch Snapshot Research Group which was described in detail previously.^14^ All 71 participating hospitals retrospectively added long term follow‐up data to the prospective short‐term data collected through the Dutch Surgical Colorectal Audit for all consecutive patients who underwent resection of primary rectal cancer in 2011. Only elective cases were included in the analyses since staging might not be fully completed in patients who require emergency surgery. Patients with M1 disease were excluded, as well as patients with missing or undefined cN or (y)pN category. The study was approved by the Medical Ethics Committee of the Amsterdam University Medical Center and the need for individual informed consent was waived.

### 2.1 Staging

Staging included an abdominal computed tomography (CT)‐scan, thoracic X‐ray, and in most patients a pelvic MRI. The fifth edition TNM staging was applied, because this version was used during the study period in Dutch hospitals.^15^ Based on this edition, patients were classified as N0 in the absence of lymph node metastasis, as N1 in case of up to three regional tumor positive lymph nodes, and N2 for four of more positive nodes. The national guideline‐recommended considering lymph nodes positive when their size was equal to or greater than 5 mm on MRI images. Tumor nodules in the perirectal adipose tissue without lymphatic tissue were regarded as lymph node metastases when the diameter exceeded 3 mm at pathology. Positive non‐regional lymph nodes were staged as M1 disease. The national guideline of 2008, which was still valid in 2011, recommended neoadjuvant radiotherapy for all cT2‐4 tumors, with the exception of proximal cT2N0 tumors. Chemoradiotherapy could be considered for cT3/4 and cN2 categories, while short‐course radiotherapy was recommended for the remaining patients.

### 2.2 Variables

Low anterior resection was defined as a TME with the formation of an anastomosis, with or without diverting stoma. Abdominoperineal resection was defined as a rectal resection according to TME principles including the anal sphincter complex with a permanent colostomy. Low Hartmann's procedure was defined as a (low) anterior resection with closure of the rectal stump and the formation of an end colostomy. Any disease recurrence in the pelvis, at the anastomosis, or in the perineal wound was defined as local recurrence. Recurrence at other locations not present at the time of rectal resection was defined as distant recurrence and termed metastasis‐free survival within the current study. Metastasis‐free survival and local recurrence‐free survival were defined as the time from surgery to recurrence or last follow‐up. Overall survival was defined as the time from surgery to death or last follow‐up.

### 2.3 Statistical analysis

All continuous variables were displayed as median with interquartile range, with the exception of hazard ratios for which the 95% confidence interval was reported. Categorical variables were reported as numbers with percentages and differences were tested using *χ* ^2^ tests. Survival curves were generated using the Kaplan‐Meier method, and differences between groups were tested using logrank tests. Multivariable analyses were performed using the Cox‐proportional hazard method. For local recurrence and metastasis‐free survival, the effect of clinical nodal staging was corrected for pT‐category (dichromate variable (y)pT0‐2 vs (y)pT3‐4), positive resection margin, and neoadjuvant treatment regimen. These variables were considered the main factors to influence the risk of (local) recurrence. The cohort size and number of events did not allow for the inclusion of additional variables. For overall survival, age was added to the multivariable analysis. All statistical analyses were performed using SPSS (Version 24.0; IBM, Chicago, IL) and the survival curves were plotted using GraphPad Prism (version 8.0; Graphpad Inc, La Jolla, CA).

## 3 RESULTS

In total, 2095 underwent resection for rectal cancer in 2011 in The Netherlands and were included in the snapshot study cohort. Eighty‐seven patients underwent emergency surgery and were excluded. A further 134 patients were excluded due to M1 disease. Finally, 326 patients were excluded due to either undetermined or missing cN category (n = 293) and/or pN category (n = 43). Baseline characteristics of the 1548 included patients are shown in Table 1.

**Table 1:** Patient and disease characteristics

	n = 1548
Age, median (IQR)	67 (60‐75)
Male sex, n (%)	984 (64)
ASA score, III or IV, n (%)	256 (17)
Distance to anal verge, n (%)	
<3 cm	358 (23)
3.1‐7.0 cm	445 (29)
>7 cm	423 (27)
Preoperative MRI, n (%)	1435 (93)
cT category, n (%)	
T1	72 (5)
T2	415 (27)
T3	883 (57)
T4	130 (8)
cN category, n (%)	
N0	695 (45)
N1	594 (38)
N2	259 (17)
Mesorectal margin <1 mm, n (%)	433 (28)
Neoadjuvant therapy, n (%)	
None	131 (9)
5 ×5 Gy, short interval	678 (44)
5 ×5 Gy, long interval	66 (4)
Chemoradiotherapy	525 (34)
Other	148 (10)
Procedure, n (%)	
Low anterior resection	766 (50)
Abdominoperineal resection	472 (31)
Low Hartmann	272 (18)
Other	38 (2)
Laparoscopy, n (%)	754 (49)
Negative margins, n (%)	1467 (95)
pT‐category, n (%)	
T0	108 (7)
T1	118 (8)
T2	521 (34)
T3	701 (45)
T4	66 (4)
pN category, n (%)	
N0	1025 (66)
N1	381 (25)
N2	142 (9)

Abbreviation: IQR, interquartile range.

### 3.1 Staging

Out of 695 patients (45%) who were staged cN0, 135 (19%) were postoperatively staged as (y)pN1 and 33 (5%) as (y)pN2. Out of the 594 (38%) patients staged cN1, 65 patients (11%) were staged (y)pN2 after surgery. Out of the entire cohort, 233 (15%) of patients had a higher (y)pN category than the preoperative cN category.

Upfront surgery without neoadjuvant treatment was performed in 131 patients (8%). The majority had cN0 disease (84 [64%]), of which 20 (24%) were ultimately staged pN1 and 5 (6%) pN2 (Table 2). A further 38 patients were staged cN1, of whom 5 (16%) were pN2. This resulted in 31 (24%) patients with a higher pN compared to the preoperative cN category in patients who underwent surgery alone.

**Table 2:** pathological N‐category according to clinical N‐category

	No neoadjuvant treatment		
	cN0	cN1	cN2
(y)pN0	59 (70)	16 (42)	6 (67)
(y)pN1	20 (24)	16 (42)	1 (11)
(y)pN2	5 (6)	1 (11)	2 (22)
5×5 Gy—short interval			
(y)pN0	305 (74)	126 (52)	14 (58)
(y)pN1	87 (21)	88 (36)	4 (17)
(y)pN2	19 (5)	29 (12)	6 (25)
Chemoradiotherapy			
(y)pN0	93 (84)	151 (64)	111 (63)
(y)pN1	11 (10)	68 (29)	40 (23)
(y)pN2	7 (6)	19 (8)	25 (14)

Neoadjuvant treatment consisted of short‐course radiotherapy (5 × 5 Gy) in 744 patients (48%), with a short interval to surgery in 678 patients (91%) and long interval in 66 patients (9%). Of the 411 patients with a short interval to surgery after 5 × 5 Gy and a cN0 category, 87 (21%) were ypN1 and 19 (5%) ypN2. An unexpected ypN2 category was found in 29 patients (12%) out of the 243 cN1 patients. This amounted to 135 (20%) understaged patients who underwent short‐course radiotherapy before surgery.

There was a trend towards less lymph node understaging by the use of MRI. Understaging (ie, a pN category higher than the cN category) occurred in 21 out of the 95 (22%) without a preoperative MRI, compared to 210 out of the 1435 patients (17%) having MRI (*P* = .054). For the remaining 18 patients, it was unknown whether a preoperative MRI was performed.

#### 3.1.1 Local recurrence and survival

**Figure 1:**
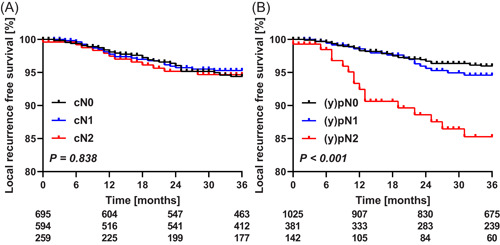
Local recurrence‐free survival according to (A) cN category and (B) (y)pN category. Curves were generated according to the Kaplan‐Meier methods, differences between groups were tested using logrank tests, and numbers of patients at risk are depicted below the graphs [Color figure can be viewed at wileyonlinelibrary.com]

Overall local recurrence rate at 36 months was 5%. These rates were similar for cN0, cN1, and cN2 staged patients, being 6%, 5%, and 5%, respectively (Figure 1A). The pathological N‐category was more predictive for local recurrence rate at 36 months: 4%, 5%, and 15% for (y)pN0, (y)pN1, and (y)pN2, respectively (Figure 1B). Local recurrence rate was 8% at 36 months after upfront surgery, 2% after short‐course radiotherapy with a short interval to surgery, and 6% after chemoradiotherapy followed by surgery.

Multivariable analysis revealed that for patients with (y)pN1 disease, short‐course radiotherapy before surgery was associated with lower local recurrence rates (Table 3). Underestimated cN category was not predictive for local recurrence. For patients with a (y)pN2‐category, multivariable analysis identified only advanced T‐category to be associated with local recurrence, and short‐course radiotherapy before surgery approached statistical significance (Table 3).

**Table 3:** Multivariable analysis for local recurrence in patients staged (y)pN1, and (y)pN2

(y)pN1	Hazard ratio (95% CI)	*P* value
Neoadjuvant therapy		
None	Reference	
Short‐course radiotherapy—short interval	0.26 (0.72‐0.95)	.042
Chemoradiotherapy	0.47 (0.12‐1.88)	.285
Other	1.49 (0.43‐5.17)	.534
Underestimated cN category, cN < (y)pN stage	1.67 (0.68‐4.12)	.263
pT‐category, pT3‐4 vs pT0‐2	1.42 (0.96‐2.10)	.075
Tumor positive resection margin	2.08 (0.65‐6.72)	.192

(y)pN2	Hazard ratio (95% CI)	*P* value
Neoadjuvant therapy		
None	Reference	
Short‐course radiotherapy—short interval	0.27 (0.08‐0.98)	.046
Chemoradiotherapy	0.39 (0.09‐1.58)	.186
Other	1.38 (0.39‐4.83)	.615
Underestimated cN category, cN < (y)pN stage	1.91 (0.75‐4.84)	.175
pT‐category, pT3‐4 vs pT0‐2	1.58 (1.15‐4.83)	.004
Tumor positive resection margin	22.21 (0.69‐7.12)	.184

Abbreviation: CI, confidence interval.

Distant metastasis‐free survival was 81% at 36 months after surgery. The rates were 84%, 79%, and 77% for cN0, cN1, and cN2, respectively, and 88%, 66%, and 62% for (y)pN0, (y)pN1, and (y)pN2, respectively (Figure SIA,B). The metastasis‐free survival was 80% for patients who underwent upfront surgery, 85% after short‐course radiotherapy with a short interval to surgery and 75% after chemoradiotherapy followed by surgery. At multivariable analysis, clinical nodal understaging had no impact on the rate of metastasis‐free survival, neither in patients staged (y)pN1, nor for those staged (y)pN2 (Table SI).

**Figure 2:**
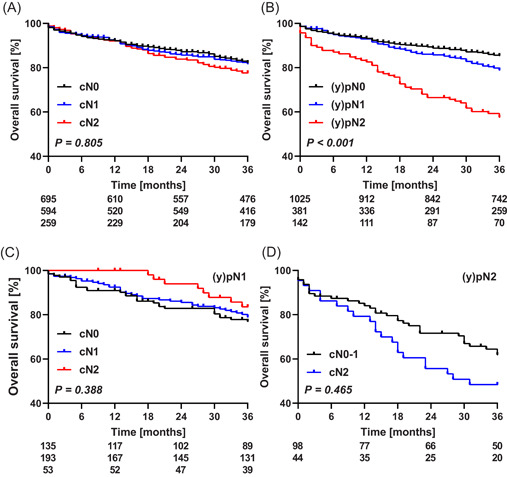
Overall survival according to (A) cN category and (B) (y)pN category. Overall survival in patients with (y)pN1 according to cN category is shown in (C) and for (y)pN2 patients in (D). Curves were generated according to the Kaplan‐Meier methods, differences between groups were tested using logrank tests, and numbers of patients at risk are depicted below the graphs [Color figure can be viewed at wileyonlinelibrary.com]

Overall survival at 3 years was 81%, which was similar for all cN categories (cN0 82%, cN1 82%, and cN2 78%), but dependent on the pN category ((y)pN0 85%, (y)pN1 79%, and (y)pN2 58%, *P* < .001) (Figure 2A,B). Three‐year overall survival after upfront surgery was 72%, which was 85% and 82% after short‐course radiotherapy and chemoradiotherapy, respectively (*P* < .001).

There was no effect of clinical nodal understaging on 3‐year overall survival in patients staged (y)pN1:77%, 79%, and 83%, for cN0, 1, and 2, respectively (*P* = .388) (Figure 2C). The majority of these patients underwent either short‐course radiotherapy (47% [179/381]) or chemoradiotherapy (31% [119/381]) before surgery, but in none of these subgroups a survival difference was found. In patients staged (y)pN2, the overall survival was similar for those staged cN0‐1 and cN2 (70% and 48%, respectively) (Figure 2D). In multivariable analysis, an underestimated cN category was not associated with overall survival, neither in (y)pN1, nor in patient staged (y)pN2 (Table SII).

## 4 DISCUSSION

In a cohort of 1548 patients who underwent surgical resection for rectal cancer in 2011 in The Netherlands, 233 patients (15%) were clinically understaged considering the actual postoperative lymph node category. Clinical lymph node understaging had no significant impact on local recurrence rates, did not impact metastasis‐free survival, and was not associated with lower overall survival rates.

Several randomized trials have shown the prognostic significance of local lymph node metastases on local recurrence rates which was confirmed in the nationwide data.^16^, ^17^, ^18^, ^19^ Two large meta‐analyses evaluated the accuracy of endoscopic ultrasound, CT, and MRI for nodal staging. One included 90 studies and found similar accuracy for all modalities, but with a limited sensitivity that did not exceed 67%.^9^ The other with 75 articles found a slight but nonsignificant advantage for endoscopic ultrasound.^20^ The reported sensitivity for the detection of nodal disease ranged from 57% to 85%, meaning positive nodes were overlooked in one out over every two to seven patients. Size alone was insufficient to discriminate tumor positive nodes on MRI, contour and signal intensity have been shown to increase discrimination.^21^, ^22^, ^23^ Up to 58% of positive nodes were less than 5 mm, negative nodes more than 10 mm were not uncommon and in patients with positive nodes concurrent reactive nodes were often of similar or even greater size.^21^, ^22^ Combining size with the morphological criteria increased the accuracy but to the limited extent mentioned above.

In The Netherlands, nodal status is one of the criteria for the selection of patients for the most appropriate neoadjuvant treatment regimen. Almost all randomized rectal cancer trials on neoadjuvant treatment did not incorporate routine MRI staging. Several studies have reported that node positive disease was a risk factor for local recurrence.^10^, ^24^ The Dutch TME trial showed that short‐course radiotherapy reduced local recurrence at 2 years by over threefold at univariable analysis.^10^ A similar Swedish trial also revealed a twofold reduction in local recurrence by preoperative short‐course radiotherapy in node positive disease.^25^ These results indicated that patients understaged as cN1 at preoperative MRI and who did not undergo short‐course radiotherapy were at increased risk of local recurrence. Whether MRI staging can improve risk stratification beyond the clinical staging as used in these previous trial remains to be investigated in future trials. Furthermore, quality of TME surgery has significantly improved over time, which has diminished the absolute risk reduction that can be obtained by adding neoadjuvant radiotherapy. The overall rate of positive resection margins in the TME trial was 15%, while the current rate of incomplete resection is below 10% overall, and below 5% for early to intermediate risk rectal cancer without neoadjuvant radiotherapy.^26^, ^27^, ^28^

Patients with a preoperative underestimated lymph node stage did not have inferior local recurrence rates or overall survival. This observation leaves open to the current use of preoperative nodal category to select patients for neoadjuvant treatment. Especially considering that in other countries such as the UK, lymph node status does not dictate neoadjuvant treatment.^29^ With more accurate diagnostic imaging and more reliable preoperative identification of positive lymph nodes, the true impact of positive lymph nodes in rectal cancer might become apparent. However, considering the overall low local recurrence rate of 5% at 3 years after surgery in the current cohort, a trial to further reduced local recurrence rate will require a very large sample size. Furthermore it should be mentioned that only a small number of patients did not receive any neoadjuvant therapy. This can also contribute to the similar recurrence rates found in patients with preoperative underestimated nodal status.

A significant proportion of local recurrences arise from enlarged lateral lymph nodes, which are not completely eradicated by chemoradiotherapy and not resected with TME. Lateral lymph node dissection in case of enlarged lateral lymph nodes on a preoperative MRI could possibly help reduce local recurrence rates. Monitoring the effects in a trial on such intervention will require standardized reporting using currently absent consensus criteria for suspicious lymph nodes.^30^, ^31^ Developments in the field of MRI such as nanoparticle‐enhanced MRI can also increase the diagnostic accuracy,^32^ but eliminating subjectivity towards suspicious lymph nodes through automated analyses of MRI images using deep learning techniques should also be subject of future research.^33^

Unexpectedly positive lymph nodes suggests the use of adjuvant therapy. Adjuvant therapy after resection with positive circumferential margin seems to have no benefit.^34^ Some studies do suggest a reduction in local recurrence rates with adjuvant therapy for high‐risk patients who did not receive neoadjuvant treatment.^35^, ^36^, ^37^ However this subject is controversial, and considering the low overall local recurrence rates would result in substantial overtreatment. Due to the probable high number of patients needed to treat, the benefits are unlikely to outweigh the negative side effects of adjuvant therapy.

As the criteria for positive lymph nodes on MRI images are not unequivocal, not only understaging, but also overstaging is a problem. Although analyzing overstaging was not the aim of the analysis, Table 2 shows it does occur. Although the numbers are small, due to the neoadjuvant therapies that hamper analyses on overstaging, it is a problem discussed in literature.^38^, ^39^ Overstaging can lead to unnecessary neoadjuvant treatment that might not offer a benefit, but does do harm. Short‐course radiotherapy is associated with more frequent fecal incontinence, sexual problems, and delayed wound healing.^40^, ^41^, ^42^, ^43^

The large national cross‐sectional study design is a strength of the present study. The limitations include the retrospective data collection. Due to the voluntary participation, not all Dutch hospitals (71/94) contributed data. Nevertheless the cross‐sectional design provided data representing daily clinical practice. Also the neoadjuvant treatment regimens do not allow complete correlation of cN and pN category due to possible tumor regression. Only a minority of patients received surgery alone at the time rectal cancer was significantly overtreated with radiotherapy in the Netherlands.^44^ In the 2014 revised Dutch guideline, radiotherapy was no longer recommended for low risk rectal cancers. Furthermore, MRI criteria were introduced to assess mesorectal lymph node status. It would therefore be very insightful to repeat this analysis for current Dutch practice. Finally, other negative predictive factors that can be extracted from MRI imaging, such as extramural vascular invasion, were not available in the current dataset, while these might be able to explain some differential outcomes.^45^

In conclusion, understaging of nodal status is not uncommon but not associated with higher rates of (local) recurrence or inferior overall survival. Although the overall outcomes of the cohort were comparable to large randomized trials, future studies should assess the effect of preoperative staging accuracy on outcomes with the current more restricted neoadjuvant therapy indications.

## DUTCH SNAPSHOT RESEARCH GROUP

*Collaborators: AGJ Aalbers, Y Acherman, GD Algie, B Alting von Geusau, F Amelung, TS Aukema, IS Bakker, SA Bartels, S Basha, AJNM Bastiaansen, E Belgers, W Bleeker, J Blok, RJI Bosker, JW Bosmans, MC Boute, ND Bouvy, H Bouwman, A Brandt‐Kerkhof, DJ Brinkman, S Bruin, ERJ Bruns, JPM Burbach, JWA Burger, CJ Buskens, S Clermonts, PPLO Coene, C Compaan, ECJ Consten, T Darbyshire, SML de Mik, EJR de Graaf, I de Groot, RJ de Vos tot Nederveen Cappel, JHW de Wilt, J van der Wolde, FC den Boer, JWT Dekker, A Demirkiran, M Derkx‐Hendriksen, FR Dijkstra, P van Duijvendijk, MS Dunker, QE Eijsbouts, H Fabry, F Ferenschild, JW Foppen, EJB Furnee, MF Gerhards, P van Gerven, JAH Gooszen, JA Govaert, WMU Van Grevenstein, R Haen, JJ Harlaar, E van der Harst, K Havenga, J Heemskerk, JF Heeren, B Heijnen, P Heres, C Hoff, W Hogendoorn, P Hoogland, A Huijbers, P Janssen, AC Jongen, FH Jonker, EG Karthaus, A Keijzer, JMA Ketel, J Klaase, FWH Kloppenberg, ME Kool, R Kortekaas, PM Kruyt, JT Kuiper, B Lamme, JF Lange, T Lettinga, DJ Lips, F Logeman, MF Lutke Holzik, E Madsen, A Mamound, CC Marres, I Masselink, M Meerdink, AG Menon, JS Mieog, D Mierlo, GD Musters, GAP Nieuwenhuijzen, PA Neijenhuis, J Nonner, M Oostdijk, SJ Oosterling, PMP Paul, KCMJ Peeters, ITA Pereboom, F Polat, P Poortman, M Raber, BMM Reiber, RJ Renger, CC van Rossem, HJ Rutten, A Rutten, R Schaapman, M Scheer, L Schoonderwoerd, N Schouten, AM Schreuder, WH Schreurs, GA Simkens, GD Slooter, HCE Sluijmer, N Smakman, R Smeenk, HS Snijders, DJA Sonneveld, B Spaansen, EJ Spillenaar Bilgen, E Steller, WH Steup, C Steur, E Stortelder, J Straatman, HA Swank, C Sietses, HA Groen, HG ten Hoeve, WW ter Riele, IM Thorensen, B Tip‐Pluijm, BR Toorenvliet, L Tseng, JB Tuynman, J van Bastelaar, SC van Beek, AWH van de Ven, MAJ van de Weijer, C van den Berg, I van den Bosch, JDW van der Bilt, SJ van der Hagen, R van der Hul, G van der Schelling, A van der Spek, N van der Wielen, E van Duyn, C van Eekelen, JA van Essen, K van Gangelt, AAW van Geloven, C van Kessel, YT van Loon, A van Rijswijk, SJ van Rooijen, T van Sprundel, L van Steensel, WF van Tets, HL van Westreenen, S Veltkamp, T Verhaak, PM Verheijen, L Versluis‐Ossenwaarde, S Vijfhuize, WJ Vles, SC Voeten, FJ Vogelaar, WW Vrijland, E Westerduin, ME Westerterp, M. Wetzel, KP Wevers, B Wiering, CDM Witjes, MW Wouters, STK Yauw, ES van der Zaag, EC Zeestraten, DDE Zimmerman, T Zwieten.

## SYNOPSIS

Compared to postoperative pathology, 233 out of 1548 (15%) rectal cancer patients had a preoperatively underestimated lymph node category. Understaging was not associated with local recurrence or overall survival rates.

## Supporting information

Supporting information

## Data Availability

The data that support the findings of this study are available on request from the corresponding author. The data are not publicly available due to privacy or ethical restrictions.
