# Supplementary material for: Effect of understaging on local recurrence of rectal cancer
Source: J Surg Oncol. 2020 Jul 11;122(6):1179–86. doi: 10.1002/jso.26111 (PMC7689834; doi:10.1002/jso.26111)
Supplement: Supplementary file 1 — Supporting information [file JSO-122-1179-s001.docx]

SUPPLEMENTAL MATERIAL

**Effect of understaging on local recurrence of rectal cancer**

Louis J.X. Giesen MD^1^, Wernard A. A. Borstlap MD PhD^2^, Willem A. Bemelman MD PhD^2^, Pieter J. Tanis MD PhD^2^, Cornelis Verhoef MD PhD^1^, Pim B. Olthof MD PhD^1^, *Dutch Snapshot Research Group*

*^1^Department of Surgical Oncology, Erasmus MC Cancer Institute, Rotterdam, the Netherlands
^2^Department of Surgery, Amsterdam UMC, University of Amsterdam, Cancer Centre Amsterdam, Amsterdam, the Netherlands*

*Dutch Snapshot Research Group:* *Collaborators: AGJ Aalbers , Y Acherman, GD Algie, B Alting von Geusau, F Amelung, TS Aukema, IS Bakker, SA Bartels , S Basha, AJNM Bastiaansen, E Belgers, W Bleeker, J Blok, RJI Bosker, JW Bosmans, MC Boute, ND Bouvy, H Bouwman, A Brandt-Kerkhof, DJ Brinkman, S Bruin, ERJ Bruns, JPM Burbach, JWA Burger, CJ Buskens, S Clermonts, PPLO Coene, C Compaan, ECJ Consten, T Darbyshire, SML de Mik , EJR de Graaf, I de Groot, RJ de Vos tot Nederveen Cappel, JHW de Wilt, J van der Wolde, FC den Boer, JWT Dekker, A Demirkiran, M Derkx-Hendriksen, FR Dijkstra, P van Duijvendijk, MS Dunker, QE Eijsbouts, H Fabry, F Ferenschild, JW Foppen, EJB Furnee, MF Gerhards, P van Gerven, JAH Gooszen, JA Govaert, WMU Van Grevenstein, R Haen, JJ Harlaar , E van der Harst, K Havenga, J Heemskerk, JF Heeren, B Heijnen, P Heres, C Hoff, W Hogendoorn, P Hoogland, A Huijbers,  P Janssen, AC Jongen, FH Jonker, EG Karthaus, A Keijzer, JMA Ketel, J Klaase, FWH Kloppenberg, ME Kool, R Kortekaas, PM Kruyt, JT Kuiper, B Lamme, JF Lange, T Lettinga, DJ Lips, F Logeman, MF Lutke Holzik, E Madsen, A  Mamound, CC Marres, I Masselink, M Meerdink, AG Menon, JS Mieog, D Mierlo, GD Musters, GAP Nieuwenhuijzen, PA Neijenhuis, J Nonner, M Oostdijk, SJ Oosterling, PMP Paul, KCMJ Peeters, ITA Pereboom, F Polat, P Poortman, M Raber, BMM Reiber, RJ Renger, CC van Rossem, HJ Rutten, A Rutten, R Schaapman, M Scheer, L Schoonderwoerd, N Schouten, AM Schreuder, WH Schreurs, GA Simkens, GD Slooter, HCE Sluijmer, N Smakman, R Smeenk, HS Snijders, DJA Sonneveld, B Spaansen, EJ Spillenaar Bilgen, E Steller, WH Steup, C Steur, E Stortelder, J Straatman, , HA Swank, C Sietses, HA Groen, HG ten Hoeve, WW ter Riele, IM Thorensen, B Tip-Pluijm, BR  Toorenvliet, L Tseng, JB Tuynman, J van Bastelaar, SC van Beek, AWH van de Ven, MAJ van de Weijer, C van den Berg, I van den Bosch, JDW van der Bilt, SJ van der Hagen, R van der Hul, G van der Schelling, A van der Spek, N van der Wielen, E van Duyn, C van Eekelen, JA van Essen, K van Gangelt, AAW van Geloven, C van Kessel, YT van Loon, A van Rijswijk, SJ van Rooijen, T van Sprundel, L van Steensel, WF van Tets, HL van Westreenen, S Veltkamp, T Verhaak, PM Verheijen, L Versluis-Ossenwaarde, S Vijfhuize, WJ Vles, SC Voeten, FJ Vogelaar, WW Vrijland, E Westerduin, ME Westerterp, M. Wetzel, KP Wevers, B Wiering, CDM Witjes, MW Wouters, STK Yauw, ES van der Zaag, EC Zeestraten, DDE Zimmerman , T Zwieten

Running head: Understaging of rectal cancer

The authors report no conflicts of interest.

**Corresponding author:**Pim B. Olthof, MD PhD
Department of Surgery
Erasmus Medical Center
Doctor Molewaterplein 40
3015 GD Rotterdam
The Netherlands
p.olthof@erasmusmc.nl

**Table SI:** Multivariable analysis for distant metastases free survival survival according to (y)pN subgroups.

| **(y)pN1** | **Hazard ratio (95%CI)** | **P-value** |
| --- | --- | --- |
| **Neoadjuvant therapy**  *None*  *Short-course radiotherapy – short interval*  *Chemoradiotherapy*  *Other* | reference 0.88 (0.41-1.88) 1.73 (0.80-3.77) 1.14 (0.47-2.75) | 0.743 0.165 0.778 |
| **Underestimated cN-category,** *cN < (y)pN category* | 0.71 (0.45-1.12) | 0.137 |
| **pT-category***, pT3-4 versus pT0-2* | 1.26 (1.06-1.49) | 0.008 |
| **Tumor positive resection margin** | 1.05 (0.48-2.33) | 0.896 |
|  | | |
| **(y)pN2** | **Hazard ratio (95%CI)** | **P-value** |
| **Neoadjuvant therapy**  *None*  *Short-course radiotherapy – short interval*  *Chemoradiotherapy*  *Other* | reference 1.07 (0.35-3.23) 1.69 (0.57-4.99) 0.76 (0.20-2.92) | 0.909 0.347 0.693 |
| **Underestimated cN-ctagory,** *cN < (y)pN stagory* | 0.79 (0.42-1.50) | 0.467 |
| **pT-category***, pT3-4 versus pT0-2* | 12.24 (1.67-89.50) | 0.014 |
| **Tumor positive resection margin** | 1.86 (0.80-4.30) | 0.148 |

**Table SII:** Multivariable analysis for overall survival according to (y)pN subgroups.

| **(y)pN1** | **Hazard ratio (95%CI)** | **P-value** |
| --- | --- | --- |
| **Neoadjuvant therapy**  *None*  *Short-course radiotherapy – short interval*  *Chemoradiotherapy*  *Other* | reference 0.64 (0.34-1.20) 0.98 (0.47-2.06) 0.93 (0.44-1.95) | 0.164 0.967 0.846 |
| **Underestimated cN-category,** *cN < (y)pN category* | 1.04 (0.65-1.65) | 0.884 |
| **pT-category***, pT3-4 versus pT0-2* | 1.19 (0.93-1.08)) | 0.163 |
| **Tumor positive resection margin** | 1.98 (1.10-3.58) | 0.024 |
| **Age***, continuous* | 1.06 (1.03-1.08) | 0.163 |
|  | | |
| **(y)pN2** | **Hazard ratio (95%CI)** | **P-value** |
| **Neoadjuvant therapy**  *None*  *Short-course radiotherapy – short interval*  *Chemoradiotherapy*  *Other* | reference 0.79 (0.33-1.89) 1.16 (0.47-2.87) 0.91 (0.34-2.40) | 0.590 0.749 0.844 |
| **Underestimated cN-category,** *cN < (y)pN category* | 0.80 (0.44-1.45) | 0.455 |
| **pT-category***, pT3-4 versus pT0-2* | 3.61 (1.29-10.09) | 0.015 |
| **Tumor positive resection margin** | 2.03 (1.06-3.87) | 0.033 |
| **Age***, continuous* | 1.04 (1.02-1.07) | 0.001 |

**Figure SI**: Metastases free survival according to (**A**) cN category and (**B**) (y)pN category. Curves were generated according to the Kaplan-Meier methods, differences between groups were tested using log-rank tests, and numbers of patients at risk are depicted below the graphs.
